# A new case of Echovirus 11 neonatal fulminant hepatitis involving male twins in a Northern Italy Tertiary University Hospital: Insight on a possible immunological clue

**DOI:** 10.1016/j.ijregi.2024.100411

**Published:** 2024-07-26

**Authors:** Federica Novazzi, Antonio Piralla, Simona Perniciaro, Angelo Paolo Genoni, Francesca Drago Ferrante, Federica Giardina, Guglielmo Ferrari, Laura Pellegrinelli, Nicola Clementi, Elena Pariani, Ivan Zanoni, Fausto Baldanti, Massimo Agosti, Nicasio Mancini

**Affiliations:** 1 University of Insubria, Department of Medicine and Technological Innovation Varese, Varese, Italy; 2 ASST Sette Laghi, Laboratory of Medical Microbiology and Virology, Varese, Italy; 3 Fondazione IRCCS Policlinico San Matteo, Microbiology and Virology Department, Pavia, Italy; 4 Ospedale Filippo del Ponte Ostetricia e Ginecologia, Neonatology and NICU, Del Ponte Hospital, Varese, Italy; 5 University of Pavia, Department of Clinical, Surgical, Diagnostic and Pediatric Sciences Pavia, Pavia, Italy; 6 University of Milan, Department of Biomedical Sciences for Health Milano, Milano, Italy; 7 IRCCS San Raffaele Hospital, Milan, Italy, Department of Biomedical Sciences for Health, Milano, Italy; 8 Harvard University, Boston Children's Hospital Cambridge, Cambridge, USA; 9 University of Insubria, Department of Medicine and Surgery, Varese, Italy

**Keywords:** Echovirus 11, Enterovirus, Hepatitis, Neonatal infection, Mucosal immunity

## Abstract

The European Center for Disease Prevention and Control has reported 19 cases of severe echovirus 11 infections in neonates since 2022, nine of which were fatal. We report a new fatal neonatal case that occurred in a male twin for which we evaluated the respiratory and intestinal mucosal innate immune response.

## Highlights

• Echovirus 11 is still circulating in Italy and can be fatal in newborns.
• Our report confirms Echovirus 11 deserves attention to control and limit its spread.
• A massive local interferon response was observed in the newborn.

## Introduction

Infections caused by enteroviruses (EV) are a significant warning in the neonatal disease burden and public health [[1], [2], [3], [4], [5]]. The main clinical signs of EV infections are fever, lethargy, and lack of appetite. EV infection usually resolves without complications. However, in a small percentage of cases, it can cause serious diseases such as hepatitis [[1], [2], [3], [4], [5]]. In this report, we describe a fatal neonatal case of congenital echovirus 11 (EV11) infection and the mucosal expression of innate immune mediators in the respiratory and intestinal mucosa of the infected twins.

## Case series

### Case description and microbiological investigation

In December 2023, two male dizygotic twins (T1 and T2) were born to a healthy mother at 35 weeks of gestation by cesarean section at the University Hospital of Varese (Italy). T1 was born with a birth weight of 2.710 g and an Apgar score of 10 at 5 minutes but, after 1 week, was transferred to the neonatal intensive care unit (NICU) and intubated because of respiratory distress and meningeal signs. In contrast, T2 (birth weight: 2.000 g; Apgar score 9 at 5 minutes) did not show any clinical signs of infection.

The clinical course of T1 was complicated by coagulopathy and thrombocytopenia requiring plasma and platelet concentrates infusion. X-ray done showed bi-basal accentuation of lung interstitium compatible with transient tachypnea of the newborn. Clear laboratory signs of massive hepatic involvement were reported (aspartate transaminase >7000 mU/ml, alanine transaminase 729 mU/ml) with concurrent multi-organ involvement (troponin 279 ng/l, creatinine 1.27 mg/dl). Cerebrospinal fluid (CSF) and bronchoalveolar lavage (BAL) were sent to the laboratory for culture-based and molecular assays. CSF white blood cell count was 20 cells/μl, protein concentration was 61 mg/dl and CSF glucose was 65 mg/dl. CSF was tested using the FilmArray Meningitis/Encephalitis panel (BioMerieux, Marcy l’Étoile, France) detects 14 of the most common pathogens in encephalitis, including bacteria, fungi and viruses, while the BAL was processed with the FilmArray Respiratory Panel 2 plus panel (BioMerieux, Marcy l’Étoile, France) recognizing 34 targets of which certain antimicrobial resistance genes as well as the most common causes of bacterial, viral, and yeast pneumonia.

Both samples tested positive for EV. The clinician in charge was immediately notified and EV genotyping was performed. Antibiotic treatment with ceftazidime and vancomycin plus antiviral acyclovir was started. EV RNA was also detected in rectal swab (RS) and plasma from T1 using the quantitative EV ELITe MGB reverse transcription-polymerase chain reaction test (ELITech Group, Turin, Italy) featuring EV RNA viral load of 83.803 copies/mL (corresponding to cycle threshold [CT] 28) and 676.241 copies/ml (CT 25), respectively. Interestingly, at the respiratory level, a preferential involvement of the lower tract was observed in T1, featuring a negative nasal swab (NS) notwithstanding the strongly positive BAL (2.245.180 copies/ml - CT 22).

NS and RS samples were collected also from T2, despite the absence of any sign of ongoing infection. Both samples tested positive, featuring 5.557 copies/ml (CT 32) in RS and 1.708.215 copies/ml (CT 24) in NS. The asymptomatic mother (M) of T1 and T2 was also tested for EV at the nasal, rectal, and vaginal (VS) level, resulting negative in NS but positive in both RS (693 copies/ml; CT 35) and VS (<500 copies/ml; CT 38).

At the same time, EV surveillance was initiated in the NICU, including 24 NS and 24 paired RS from each little inpatient and 28 NS from healthcare workers. Two newborns (N1 and N2) admitted to the NICU tested positive for low-grade EV (<500 copies/ml - CT 38) in the NS, whereas none of the NS from healthcare workers was positive. Neither N1 nor N2 showed any clinical sign of infection and both tested negative within a few days.

## Methods

### Enterovirus molecular subtyping

EV genotyping was performed by Sanger sequencing of conserved genomic regions (5′UTR, 2 C, 3D^pol^) [6] on all EV-positive samples, including CSF, BAL, RS, and blood for T1, NS and RS for T2, RS and VS for M, and NS for N1 and N2. All samples were positive for E11 featuring a very high nucleotide identity with the strains described in France (97.73%) and in Italy (97.28%). The E11-positive samples were sent to the regional reference laboratories (Fondazione IRCCS Policlinico San Matteo, Pavia and The Department of Biomedical Sciences for Health, University of Milan) for whole genome sequencing, (accession numbers PP498690 and PP498691), evidencing an average nucleotide identity of 98.9% with other Italian strains [5].

### Evaluation of respiratory and rectal mucosal innate immune response

It is well known that a balanced mucosal innate immune response is fundamental to properly control viral replication and prevent more severe complications, as repeatedly confirmed by the most severe COVID-19 cases driven by SARS-CoV-2 infection [[7], [8], [9], [10], [11]]. Based on this evidence, we assessed the levels of a panel of innate immune mediators in the respiratory and intestinal mucosa. In particular, RS and NS, or BAL for T1, were utilized to assess multiple inflammatory mediators by quantitative polymerase chain reaction. Based on our previous studies in patients with severe COVID-19 [9], we included both antiviral (interferon beta-1 [IFN-B1] and IFN lambda -1 and -2 [IFNL1 and IFNL2]) and pro-inflammatory (interleukin-1-beta [IL1B] and IL-6) molecules in our analysis. The level of each cytokine was then compared to the local E11 load for all the positive newborns described in our report (T1, T2, N1, and N2).

At the respiratory level, the expression of both antiviral and pro-inflammatory cytokines was apparently driven by E11 replication and local viral load (Figure 1a).

**Figure 1(a):**
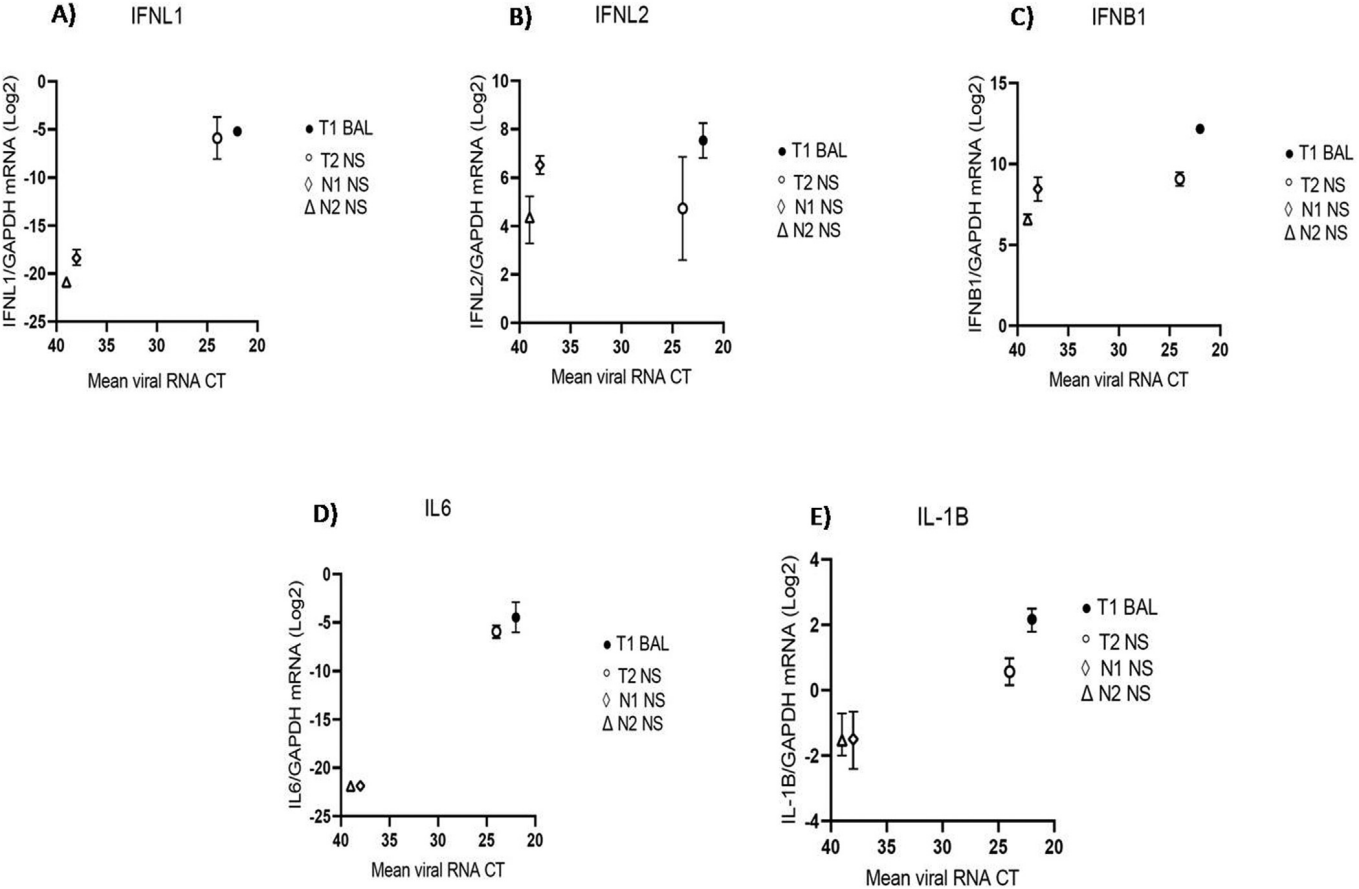
The mucosal expression of innate immune mediators parallels EV load in respiratory samples of infected newborns.

IFNL1 (A), IFNL2 (B), IFNB1 (C), IL 6 (D), and IL1B (E) mRNA expression was evaluated in respiratory samples of EV-positive newborns described in this report. Each symbol represents a newborn. The median with range is depicted. Cytokine mRNA expression is plotted against mean viral RNA CT as log2 (gene/GAPDH mRNA + 0.5 x gene-specific minimum).

Interestingly, an opposite trend was observed when analyzing the RS, in which we found very low cytokine levels in T1, the newborn with the highest E11 load, which was also the patient experiencing the worst outcome (Figure 1b).

**Figure 1(b):**
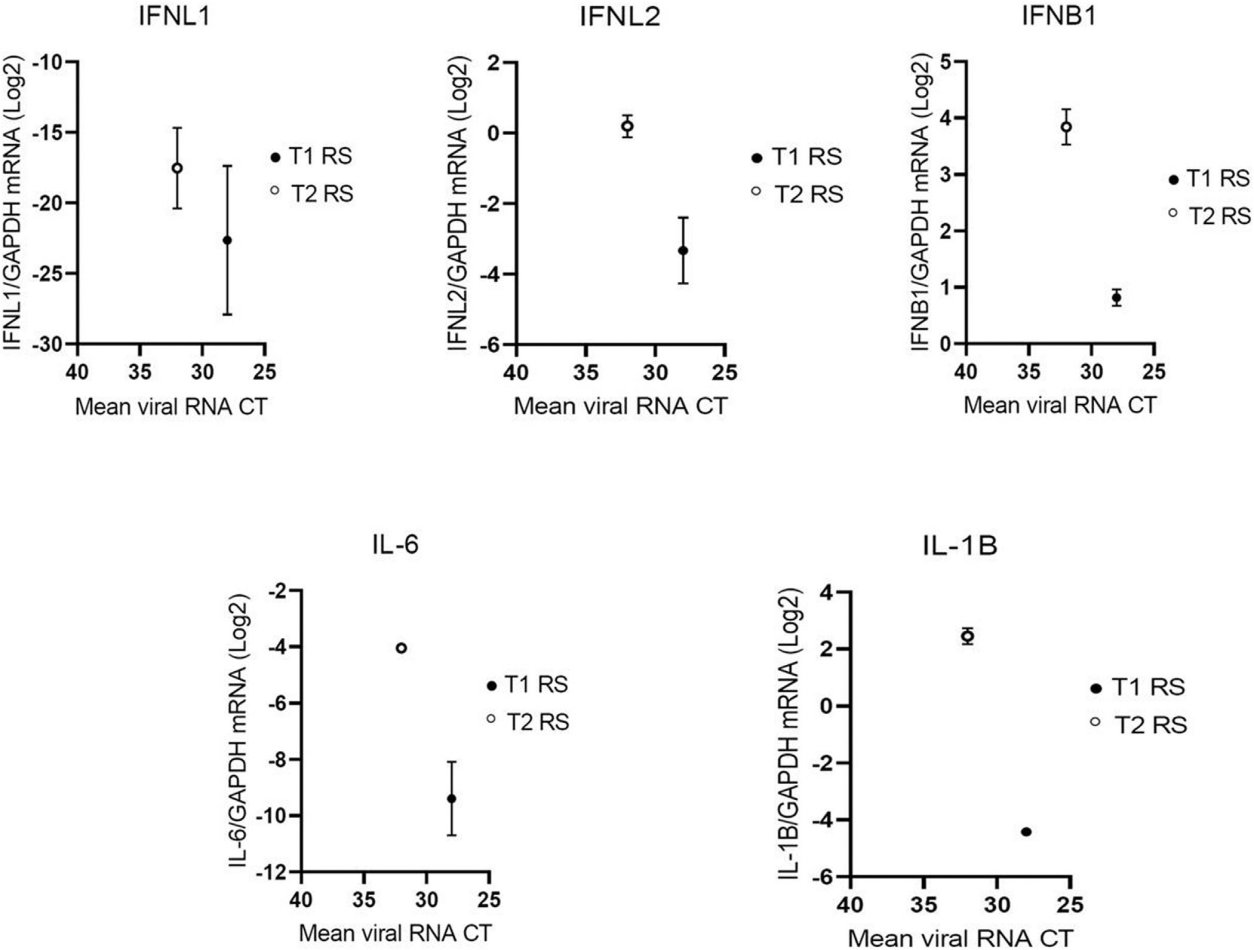
The mucosal expression of innate immune mediators is lower in the newborn with a fatal outcome, suggesting the ineffective primary control of EV replication at the gastrointestinal level.

IFNL1 (A), IFNL2 (B), IFNB1 (C), IL 6 (D), and IL1B (E) mRNA expression was evaluated in rectal swabs of EV-positive newborns described in this report. Each symbol represents a newborn. The median with range is depicted. Cytokine mRNA expression is plotted against mean viral RNA CT as log2 (gene/GAPDH mRNA + 0.5 x gene-specific minimum). Only positive EV-11 positive rectal swabs were reported. BAL, bronchoalveolar lavage; CT, cycle threshold; EV, enterovirus; GAPDH, Glyceraldehyde 3-phosphate dehydrogenase; IFN, interferon; IL, interleukin; mRNA, messenger RNA; NS, nasal swab; RS, rectal swab.

## Discussion

EV, including echoviruses such as E11, are a common cause of pediatric infections often with seasonal occurrence and a self-limiting clinical evolution, although newborns may present a higher risk of severe complications. Recently, fatal neonatal cases with massive liver failure caused by a new variant of E11 have been described, with a peculiar involvement of male non-homozygotic twins [2,3]. Our report confirms that this variant is still circulating in Europe and deserves attention to control and limit its spread. No specific evidence regarding viral factors justifying the increased pathogenicity of the new E11 variants has been described to date but, among other factors, the severity of the infection may also be determined by a differential immune control of the viral replication at the mucosal level. Local viral replication may then influence the risk of systemic spread. Most EVs infect preferentially the gastrointestinal tract (and/or the upper respiratory tract) with a possible subsequent spread to several organs, such as the central nervous system, the heart, or, as in the case of E11, the liver [10]. In the reported cases, we observed a massive local interferon response both in the airways and intestine in the newborn with no clinical complications. On the contrary, the sibling undergoing the fatal outcome was characterized by a potent antiviral response in the airways, but by a very low response at the gastrointestinal level associated with a very high viral load in the rectum. We, thus, speculate that the lack of viral control in the intestinal mucosa is a key factor favoring the systemic dissemination of E11 and, possibly, its massive spread from the gut to the liver and to other organs.

## Conclusion

As also recently described by ongoing surveillance [4,5], our report confirms that E11 is still circulating in Italy and can be fatal in newborns. Awareness must be maintained via control and prevention measures to minimize the spread of EV infections in neonatal units. The elements leading to the increased pathogenicity of the circulating E11 strain are not clear yet but, beyond still unidentified viral factors, host-related factors certainly deserve attention. No definitive conclusions may be drawn by our report, but the low levels of the antiviral innate immune response observed at the gastrointestinal level in the newborn with the fatal outcome support the hypothesis of viral spread from mucosal tissue to other organs, starting from the liver. Multi-organ involvement upon E11 infection, and stochastically upon EV infection, is associated with severe cases and the factors favoring it warrant to be studied in larger cohorts. These observations may strengthen the importance of several proposed antiviral approaches (potentially including all viruses with a pivotal phase of mucosal replication) focused on the importance of potentiating the local antiviral innate containment in at-risk categories of patients [11].

## Declarations of competing interest

The authors have no competing interests to declare.
